# Prevalence of self-medication with antibiotics and its related factors among Chinese residents: a cross-sectional study

**DOI:** 10.1186/s13756-021-00954-3

**Published:** 2021-06-05

**Authors:** Xiaoxv Yin, Ketao Mu, Heping Yang, Jing Wang, Zhenyuan Chen, Nan Jiang, Fengjie Yang, Guopeng Zhang, Jianxiong Wu

**Affiliations:** 1 Department of Social Medicine and Health Management, School of Public Health, Tongji Medical College, Huazhong University of Science and Technology, Wuhan, 430030 Hubei People’s Republic of China; 2 Department of Radiology, Tongji Hospital, Tongji Medical College, Huazhong University of Science and Technology, Jie Fang Avenue 1095, Wuhan, 430030 People’s Republic of China; 3 Wuchang University of Technology, Wuhan, Hubei People’s Republic of China; 4 Department of Pediatrics, Tongji Hospital, Tongji Medical College, Huazhong University of Science and Technology, Wuhan, 430030 Hubei People’s Republic of China; 5 Department of Nuclear Medicine, Tongji Hospital, Tongji Medical College, Huazhong University of Science and Technology, Wuhan, People’s Republic of China

**Keywords:** Antimicrobial stewardship, Antibiotic knowledge, China, Health belief model, Self-medication

## Abstract

**Background:**

Self-medication is one of the most common forms of inappropriate use of antibiotics. This study aimed to assess the prevalence of self-medication with antibiotics (SMA) in China and evaluate the related factors.

**Methods:**

A cross-sectional survey was conducted in Wuhan, Hubei, China from July 1, 2019 to July 31, 2019. Participants were recruited in public places to answer a structured questionnaire. The information of participants’ social demographic characteristics, antibiotic knowledge and health beliefs were collected. Binary Logistics regression analysis was used to examine the associated factors of SMA.

**Results:**

Of the 3206 participants, 10.32% reported SMA in the past 6 months. Participants who with middle or high perceived barriers to seek health care services showed a higher likelihood of SMA (*P* < 0.05). Participants who with middle or high perceived threats of self-medication, and who with middle or high self-efficacy to overcome obstacles showed a lower likelihood of SMA (*P* < 0.05).

**Conclusions:**

Compared with developed countries, the prevalence of SMA in China is still higher. Measures to conduct public health education and improve the accessibility of health services are crucial to decrease the overall self-medication rate in China.

**Supplementary Information:**

The online version contains supplementary material available at 10.1186/s13756-021-00954-3.

## Introduction

Under the combined action of biological and social factors, antimicrobial resistance (AMR) has become a serious threat to global public health [1, 2]. AMR may lead to more than 100 trillion dollars in economic losses by 2050 [3]. Self-medication is one of the most prevalent form of inappropriate use of antibiotics, which is based on the self-judgment of patients but lack the professional diagnosis of doctors. Such irrational behaviour may lead to negative results, such as increased risk of AMR, failure of treatments and death of the patients [4].

The global self-medication with antibiotics (SMA) reaches alarmingly high rates, especially in low-and middle-income countries [5]. A study in Tanzania showed that 58.0% of the respondents admitted to SMA (SMA) [6]. Another Serbian study found that 27.17% of the households used antibiotics without prescriptions [7]. The related factors of SMA can be divided into external factors and internal factors. The sales of antibiotics without prescriptions may be an important external factor [8], which is intensely shared in low- and middle-income countries. Although most countries had issued legislations to against these practices, they had not been effectively implemented [9, 10]. In terms of internal factors, previous studies found that age, income, education level and antibiotic knowledge were significantly correlated with self-medication [11–13].

The prevalence of SMA in China is also relatively high [14]. China's State Food and Drug Administration conducted a survey of 7915 residents, among whom 23.9% indicated that they would take antibiotics by themselves instead of seeing a doctor when they have a cold [15]. Another study conducted in rural areas of China found that 46.3% of villagers had experienced SMA [16]. A large number of studies have confirmed that the availability of over-the-counter antibiotics in China is very high [15, 17]. Most Chinese residents still have easy access to non-prescription antibiotics, which may exacerbate SMA. However, there is little research on internal factors in China. Most of the relevant studies mainly focused on college students, teenagers or children [18, 19], but rarely involved the whole population. In addition, previous studies mainly focused on the relationship between demographic characteristics, antibiotic knowledge and SMA, while ignoring the role of health beliefs. Health beliefs may have a more direct impact on behaviours, which have been widely concerned in studies on health behaviours such as breast cancer screening and hypertension self-management [20, 21].

Thus, the aims of this study were to assess the prevalence of SMA among Chinese residents and identify the associated factors.

## Methods

### Setting and participants

A cross-sectional study was conducted in Wuhan, Hubei, China, from 1 July, 2019 to 31 July, 2019. Trained investigators recruited respondents in public places such as bus stops and parks by convenient sampling method. The inclusion criteria of the subjects were: (1) adults aged 18 and older; (2) those who were able to read and write Chinese. During the data collection period, a total of 4832 questionnaires were distributed, and 3420 respondents completed the questionnaire, with a response rate of 70.78%. Among them, 129 questionnaires were deemed invalid because more than 25% of the items were missing, and 85 questionnaires were excluded because the respondents were younger than 18 years old. Finally, 3206 qualified questionnaires were included in the analysis.

### Measurement

The main outcome of this study was the prevalence of SMA, measured by asking "Did you use antibiotics without seeing a doctor in the past 6 months?" In order to identify the relevant factors of SMA, this study also collected the social demographic characteristics (gender, age, residence, educational level, self-perceived economic status, self-perceived health status), antibiotic knowledge, health beliefs of SMA.

A self-designed scale was used to measure the health beliefs of SMA. The scale is based on health belief model, including four dimensions: perceived threats, perceived benefits, perceived barriers and self-efficacy. Perceived threats refer to the degree of perceived harm caused by SMA. Perceived benefits refer to the perceived benefit of taking antibiotics under the guidance of a doctor. Perceived barriers refer to the perception of difficulty in taking antibiotics under the guidance of a doctor. Self-efficacy is the determination to overcome obstacles to seeking medical care and to take antibiotics under the guidance of a doctor. The overall Cronbach's α coefficient of the scale was 0.80. The Cronbach's α coefficients of each dimension are as follows: perceived threats (0.89), perceived benefits (0.94), perceived barriers (0.80), self-efficacy (0.84). There are 4 items of perceived threats and 5 items of perceived benefits, perceived barriers and self-efficacy respectively. Each item used Likert’s 5-point scoring method, from "totally disagree" to "totally agree", scoring 1–5 points respectively. According to the lower quartile and the upper quartile of the score, the participants' health beliefs were divided into three groups: high, middle and low.

The participants' antibiotic knowledge was measured by an Antibiotic Knowledge Scale developed by our group. The scale included 10 items with the Cronbach's α coefficient of 0.72. Each knowledge item was given a score of one point if the participant answered correctly, and a score of zero if the answer was wrong or "I don’t know". So, the total score of this scale ranged from 0 to 10. The higher the score, the better the antibiotic knowledge of participants. According to the lower quartile and the upper quartile of the score, the participants' antibiotic knowledge level was divided into three groups: high, middle and low.

### Statistical analysis

We used SPSS version 22.0 for Windows (IBM Corp., Armonk, NY, USA) for all analysis (see Additional file 1: Appendix S1 for the statistical analysis process). Descriptive statistical methods such as frequency, percentage, mean and standard deviation (SD) were used to present demographic characteristics, antibiotic knowledge, health belief and other variables. Pearson Chi-Square test was used to compare the prevalence of self-medication among participants with different characteristics. Binary Logistics regression model was used to explore the related factors of SMA. Adjusted odd ratios (ORs) and 95% confidence intervals (CIs) for each variable were given. Possible multicollinearity of variables in the model was assessed by calculating variance inflation factors (VIFs) and pairwise correlation coefficient (see Additional file 2: Appendix S2 for the multicollinearity text). Differences were tested by using two-tailed tests. If *P* < 0.05, the result was considered statistically significant.

## Results

### General characteristics and SMA of participants

Table 1 shows the demographic characteristics differences between groups defined by SMA. In the past 6 months, 10.32% of participants (331/3206) reported SMA. The majority of the participants were women (66.50%), with an average age of 33.41 ± 10.83 years old. Nearly 80% lived in cities, and more than half of the participants had college degrees or above. In addition, 73.74% rated their economic status as average, and 52.74% rated their health status as good. Furthermore, 48.60% reported that they stored antibiotics at home for use.

**Table 1:** Participants’ characteristics and associations with SMA N (%)

Variables	Number	Antibiotics self-medication		*χ*^2^	*P*
		Yes	No		
Gender				3.013	0.083
Male	1074 (33.50)	125 (11.64)	949 (88.36)		
Female	2132 (66.50)	206 (9.66)	1926 (90.34)		
Age				10.023	0.018
≤ 24	759 (23.67)	64 (8.43)	695 (91.57)		
25–44	1859 (57.99)	188 (10.11)	1671 (89.89)		
45–59	528 (16.47)	73 (13.83)	455 (86.17)		
≥ 60	60 (1.87)	6 (10.00)	54 (90.00)		
Location				5.497	0.019
Urban	2470 (77.04)	272 (11.01)	2198 (88.99)		
Rural	736 (22.96)	59 (8.02)	677 (91.98)		
Education level				7.982	0.046
Junior high school and below	470 (14.66)	40 (8.51)	430 (91.49)		
High school	703 (21.93)	76 (10.81)	627 (89.19)		
Undergraduate	1475 (46.01)	141 (9.56)	1334 (90.44)		
Postgraduate and above	558 (17.40)	74 (13.26)	484 (86.74)		
Self-perceived health status				8.480	0.014
Good	1691 (52.74)	155 (9.17)	1536 (90.83)		
Average	1334 (41.61)	148 (11.09)	1186 (88.91)		
Poor	181 (5.65)	28 (15.47)	153 (84.53)		
Self-perceived economic status				10.800	0.005
Good	452 (14.10)	66 (14.60)	386 (85.40)		
Average	2364 (73.74)	231 (9.77)	2133 (90.23)		
Poor	390 (12.16)	34 (8.72)	356 (91.28)		
Reserve antibiotics at home					
Hardly	1228 (38.30)	187 (15.23)	1041 (84.77)	74.275	0.000
Occasionally	330 (10.29)	48 (14.55)	282 (85.45)		
Always	1648 (51.40)	96 (5.83)	1552 (94.17)		
Total	3206 (100)	331 (10.32)	2875 (89.68)		

With the self-medication as the dependent variable, Pearson chi-square test was used to analyse the statistical differences. The results showed that there were significant differences in self-medication among participants of different ages, residences, educational level, self-perceived health status and self-perceived economic status.

### Antibiotic health beliefs and antibiotic knowledge of participants

Table 2 presents the participants' health beliefs and knowledge about antibiotics. In terms of health beliefs, the average scores of perceived threats, perceived benefits, perceived barriers and self-efficacy were 14.46 ± 3.51, 20.92 ± 4.14, 13.80 ± 4.31 and 18.17 ± 3.93, respectively. The percentages of participants with high perception of health beliefs in all dimensions were as follows: perceived threats (19.68%), perceived benefits (30.75%), perceived barriers (20.02%), and self-efficacy (23.42%). The average score of antibiotic knowledge of the participants was 6.86 ± 2.27. According to the quartile, the proportion of participants with higher antibiotic knowledge level is 12.04%.

**Table 2:** Participants' antibiotic knowledge level and health beliefs N (%)

Variables	Number	Antibiotics self-medication		*χ*^2^	*P*
		Yes	No		
Perceived threats				27.936	< 0.001
High	631 (19.68)	34 (5.39)	597 (94.61)		
Middle	2076 (64.75)	223 (10.74)	1853 (89.26)		
Low	499 (15.56)	74 (14.83)	425 (85.17)		
Perceived benefits				3.343	0.188
High	986 (30.75)	88 (8.92)	898 (91.08)		
Middle	1488 (46.32)	159 (10.69)	1329 (89.31)		
Low	732 (22.83)	84 (11.48)	648 (88.52)		
Perceived barriers				37.471	< 0.001
High	642 (20.02)	99 (15.42)	543 (84.58)		
Middle	2059 (64.22)	210 (10.20)	1849 (89.80)		
Low	505 (15.75)	22 (4.36)	483 (95.64)		
Self-efficacy				31.187	< 0.001
High	751 (23.42)	43 (5.72)	708 (94.27)		
Middle	1937 (60.42)	209 (10.79)	1728 (89.21)		
Low	518 (16.16)	79 (15.25)	439 (84.75)		
Antibiotic knowledge level				0.296	0.863
High	386 (12.04)	41 (10.62)	345 (89.38)		
Middle	2306 (71.93)	234 (10.15)	2072 (89.85)		
Low	514 (16.03)	56 (10.89)	458 (89.10)		

Univariate analysis showed that there were significant differences in the prevalence of self-medication among participants with different level of perceived threats, perceived barriers and self-efficacy.

### The factors associated with SMA

Before the multivariate analysis, we performed multicollinearity test on the he independent variables. The VIFs of independent variables were less than 10 and pairwise correlation coefficients were less than 0.4, indicating multicollinearity was not observed in the model.

Table 3 presents the multivariate analysis of SMA. Participants who rated themselves as in good economy status (OR = 1.81, 95%CI 1.10–2.96), always (OR = 2.62; 95%CI 2.01–3.43) and occasionally (OR = 2.34; 95%CI 1.60–3.41) stored antibiotics at home, with middle (OR = 1.77; 95%CI 1.11–2.83) or high (OR = 2.59; 95%CI 1.56–4.29) perceived barriers showed a higher likelihood of SMA. Participants who rated themselves as in good health status (OR = 0.55; 95%CI 0.35–0.88), with middle (OR = 0.69; 95%CI 0.51–0.93) or high (OR = 0.41; 95%CI 0.26–0.66) perceived threats, and with middle (OR = 0.68; 95%CI 0.51–0.92) or high (OR = 0.47; 95%CI 0.31–0.73) self-efficacy showed a lower likelihood of SMA.

**Table 3:** Logistic regression analysis results of SMA

Variables	Adjusted OR (95%CI)	*P*
Gender (Ref = Female)		
Male	1.13 (0.88–1.45)	0.334
Age (ref ≤ 24)		
25–44	1.21 (0.88–1.66)	0.251
45–59	1.41 (0.96–2.08)	0.084
≥ 60	1.06 (0.42–2.69)	0.906
Location (ref = Village)		
Urban	1.24 (0.90–1.71)	0.196
Education level (ref = Junior high school and below)		
High school	1.28 (0.84–1.95)	0.257
Undergraduate	1.16 (0.77–1.73)	0.484
Postgraduate and above	1.49 (0.95–2.33)	0.086
Self-perceived health status (ref = Poor)		
Average	0.66 (0.41–1.07)	0.084
Good	0.55 (0.35–0.88)	0.013
Self-perceived economic status (ref = Poor)		
Average	1.22 (0.81–1.84)	0.341
Good	1.81 (1.10–2.96)	0.019
Reserve antibiotics at home (ref = Hardly)		
Occasionally	2.34 (1.60–3.41)	< 0.001
Always	2.62 (2.01–3.43)	< 0.001
Antibiotic knowledge level (ref = Low)		
Middle	0.92 (0.66–1.28)	0.621
High	0.89 (0.55–1.44)	0.637
Perceived threats (ref = Low)		
Middle	0.69 (0.51–0.93)	0.015
High	0.42 (0.26–0.66)	< 0.001
Perceived benefits (ref = Low)		
Middle	1.04 (0.76–1.41)	0.813
High	1.09 (0.76–1.58)	0.629
Perceived barriers (ref = Low)		
Middle	1.77 (1.11–2.83)	0.017
High	2.59 (1.56–4.29)	< 0.001***
Self-efficacy (ref = Low)		
Middle	0.68 (0.51–0.92)	0.012
High	0.47 (0.31–0.73)	0.001

## Discussion

This study found that 10.32% of the participants had self-medicated themselves with antibiotics in the past 6 months. The ratio is lower than that in most developing countries, such as Pakistan (45%) [22] and Ghana (40%) [23], but higher than that in some developed countries, such as the United Kingdom (5%) [24]. It may be attributable to the differences in socio-economic and the sample population. Additionally, nearly half of the participants in this study stored antibiotics at home, which is considered as the main source of antibiotics for self-use [25, 26]. The results of multivariate analysis also confirmed that participants who stored antibiotics at homes were more likely to self-medicate themselves.

Participants in this study still had some misconceptions regarding antibiotics. For instance, 72.4% believed that antibiotics can cure viral infections. Knowledge is a prerequisite for behaviour change, providing individuals with basic information for health decision-making [27, 28]. Nevertheless, no significant association was found between antibiotic knowledge and SMA in current study. Previous studies also indicated that the relationship between the improvement of knowledge and the rational use of antibiotics was not simple, and merely increasing public knowledge about antibiotics may even be counterproductive [24, 29]. Health decision-making is a complex process in which individuals weigh benefits and risks [30]. Providing accurate knowledge is not enough. The changes in individual behaviour were more driven by the perception of possible adverse consequences [31, 32]. Therefore, in addition to antibiotic knowledge, we also focused on the role of health beliefs in the behavioral process.

Our study revealed that health beliefs were closely related to SMA. Individuals' perception of risks can avoid the occurrence of improper behaviours or reduce their severity [32]. Participants with high perceived threats were less likely to report SMA. When interventions successfully change an individual's risk awareness, they usually promote the transformation of improper behaviours [33]. It is necessary to carry out national antibiotic intervention programs on the irrational use of antibiotics to help residents realize the harmfulness of SMA. Participants with high perceived barriers were more likely to report self-medication. On the contrary, the higher the self-efficacy of overcoming the obstacle, the less likely they were to report self-medication. A previous study have attributed the high prevalence of self-medication to poor access to health care [34]. In addition to carrying out extensive health education, the government should also improve the accessibility of health care services to remove barriers of behavioral change.

There was a correlation between socio-demographic variables and self-medication. However, due to the different research sites and sample groups, the existing evidence is inconsistent. There is a positive correlation between economic status and self-medication in this study, which is in accord with the findings in Jordan [11], but contrary to that of Ethiopia [12]. Participants who rated themselves as in good health were less likely to self-medicate, which was consistent with the results of an Italian study [35]. Unlike previous studies, this study found no significant association between education level and self-medication. The national antibiotic intervention program should define the target population to achieve a better intervention effect.

Based on the health belief model, our study analysed the relationship between health beliefs and SMA, which was not found in previous studies. However, this study also has some limitations. First of all, there were inherent defects of a cross-sectional study, such as recall bias and difficulty in inference of causal conclusions. It is essential to further test the conclusions of this study by prospective research. Secondly, due to the lack of a scale measuring health belief in SMA, we developed a relevant scale to measure this concept. The reliability and validity of the scale need to be further tested.

## Conclusions

Compared with developed countries, the prevalence of SMA in China is still higher. Health beliefs were significantly associated with SMA. The national antibiotic intervention program should help residents understand the harm of self-medication while publicizing antibiotic knowledge. In the meanwhile, the Chinese government should improve the accessibility of health services to reduce the external obstacles to behavior change.

## Supplementary Information

**Additional file 1:** Appendix S1 for the statistical analysis process.

**Additional file 2: ** Appendix S2 for the multicollinearity text.

## Data Availability

The datasets in the present study are accessible from the corresponding author on reasonable request.

## Abbreviations

AMR: antimicrobial resistance
SMA: self-medication with antibiotics
