# Supplementary material for: Prevalence of self-medication with antibiotics and its related factors among Chinese residents: a cross-sectional study
Source: Antimicrob Resist Infect Control. 2021 Jun 5;10:89. doi: 10.1186/s13756-021-00954-3 (PMC8180170; doi:10.1186/s13756-021-00954-3)
Supplement: Supplementary file 2 — Additional file 2: Appendix S2 for the multicollinearity text. [file 13756_2021_954_MOESM2_ESM.docx]

**Additional file 2: Appendix S2 multicollinearity text**

Table 1 Pairwise correlation matrix between health beliefs and antibiotic knowledge

|  | Perceived threats | Perceived benefits | Perceived barriers | Self-efficacy | Antibiotic knowledge |
| --- | --- | --- | --- | --- | --- |
| Perceived threats | 1 | 0.322 | -0.077 | 0.273 | 0.199 |
| Perceived benefits | 0.322 | 1 | -0.107 | 0.344 | 0.280 |
| Perceived barriers | -0.077 | -0.107 | 1 | -0.199 | 0.029 |
| Self-efficacy | 0.273 | 0.344 | -0.199 | 1 | 0.169 |
| Antibiotic knowledge | 0.199 | 0.280 | 0.029 | 0.169 | 1 |

Pairwise correlation coefficients were less than 0.4, indicating there is no collinearity among the independent variables.

Table 2 Collinearity diagnosis of independent variables

| Model | | Unstandardized Coefficients | | Standardized Coefficients | t | Sig. | Collinearity Statistics | |
| --- | --- | --- | --- | --- | --- | --- | --- | --- |
|  |  | B | Std. Error | Beta |  |  | Tolerance | VIF |
| 1 | (Constant) | .114 | .057 |  | 2.005 | .045 |  |  |
|  | Gender | -.015 | .011 | -.023 | -1.303 | .193 | .971 | 1.029 |
|  | Age1 | .013 | .008 | .029 | 1.597 | .110 | .884 | 1.131 |
|  | Location | -.016 | .013 | -.023 | -1.221 | .222 | .870 | 1.150 |
|  | Education level | .010 | .006 | .030 | 1.571 | .116 | .841 | 1.189 |
|  | Self-perceived health status | .023 | .009 | .046 | 2.568 | .010 | .920 | 1.087 |
|  | Self-perceived economic status | -.031 | .011 | -.052 | -2.811 | .005 | .855 | 1.170 |
|  | Reserve antibiotics at home | .042 | .006 | .130 | 7.432 | .000 | .967 | 1.034 |
|  | Perceived threats | -.035 | .010 | -.069 | -3.673 | .000 | .852 | 1.174 |
|  | Perceived benefits | .004 | .008 | .010 | .533 | .594 | .778 | 1.285 |
|  | Perceived barriers | .035 | .009 | .069 | 3.840 | .000 | .923 | 1.084 |
|  | Self-efficacy | -.033 | .009 | -.068 | -3.541 | .000 | .812 | 1.231 |
|  | Antibiotic knowledge | -.006 | .011 | -.010 | -.512 | .609 | .841 | 1.188 |

*Dependent Variable: SMA

The VIFs were less than 10, indicating multicollinearity was not observed in the models.
